# Changes in the Structures and Directions of Heavy Metal-Contaminated Soil Remediation Research from 1999 to 2020: A Bibliometric & Scientometric Study

**DOI:** 10.3390/ijerph18147358

**Published:** 2021-07-09

**Authors:** Dongping Shi, Chengyu Xie, Jinmiao Wang, Lichun Xiong

**Affiliations:** 1 School of Environment and Resources, Xiangtan University, Xiangtan 411105, China; shidongping@xtu.edu.cn (D.S.); csuwjm@163.com (J.W.); 2 Hunan Red Solar Photoelectricity Science and Technology Co., Ltd., Changsha 410002, China; xionglc@cs48.com

**Keywords:** heavy metal-contaminated soil, remediation research, bibliometric analysis, intellectual structure, research trend

## Abstract

The pollution of heavy metals in soil is a problem of great concern to international scholars today. This research investigates the current research activities in the field of soil heavy metal pollution remediation and discusses the current areas of research focus and development trends. We conducted a bibliometric analysis of the literature on soil heavy metal pollution remediation from 1999 to 2020. CiteSpace and Vosviewer were used to conduct document co-citation and cluster analyses on the collected data. The research was mainly carried out based on the following factors: chronological distribution, country and institution distribution, source journal analysis, keyword co-occurrence analysis, and reference co-citation analysis. China (2173, 28.64%) and the United States (946, 12.47%) are the top two countries in terms of the number of articles published, and Environmental Science and Pollution Research (384, 5.06%) and Science of the Total Environment (345, 4.55%) published the most articles. The Chinese Academy of Science (485) is the organization that has contributed the most to the total number of publications. Furthermore, based on a keyword co-word analysis with Vosviewer and CitesSpace, it was concluded that the applications of phytoremediation and biochar in the remediation of heavy metals in soil are current research hotspots. Additionally, future research should focus on repair mechanisms, the development of new repair technology and joint repair systems.

## 1. Introduction

In the process of energy utilization, heavy metals are released into the environment in the form of flue gas or slag due to the combustion of certain heavy-metal fuels and migrate in the atmosphere, water and soil, causing various types of environmental pollution, such as by fossil fuels (such as coal), heavy oil, leaded gasoline, and garbage power generation. Soil is the ultimate receptor for heavy metal pollutants. The accumulation of harmful heavy metals in the soil to a certain extent will not only lead to soil degradation and crop yield and quality decline but will also pollute the surface and groundwater through rainwater, directly causing food chain poisoning or endangering human life. In recent decades, extensive research has been conducted on soil heavy metal emissions, pollution, and remediation technologies. An overview of the repair technology of heavy metals in different regions of the world is also meaningful and helpful for researchers to determine research trends, track research hotspots, and determine research directions in the next few years [1].

Bibliometric analysis is a popular and effective method used to determine research trends and important issues based on historical publications. It has been used in many science and engineering disciplines [2,3,4,5,6,7]. The traditional bibliometric method mainly focuses on the frequency of keywords and the number of published outputs of countries, research institutions, journals and subject categories. However, this method does not fully indicate the development trends of the research field. In recent years, software has been used in bibliometric analysis to perform cluster analysis to determine the development trends of the research field [8,9,10]. Currently, there are few studies on soil heavy metal pollution remediation technology based on bibliometric analysis.

This article uses CiteSpace and Vosviewer to quantitatively investigate and visually analyze the representative figures, representative works [11,12], research hotspots and frontiers of bibliometrics through the citations and citation relationships of the literature, to uncover the knowledge structure and context of the entire discipline, and then clarify the research foundation of soil heavy metal pollution remediation and determine research hotspots [13,14].

This paper analyses articles on soil heavy metal pollution remediation for the period 1999–2020. This time period was chosen because the number of relevant articles was relatively small, with fewer than 100 articles before 1999 identified through relevant literature searching and data analysis. In addition, due to the long period of time for literature publication, the final time period for analysis was 1999–2020.

## 2. Materials and Methods

### 2.1. Data Sources

In this paper, the data source is determined by subject word searches. The data analyzed are based on SCIE in the Web of Science Core Collection database of the Institute for Scientific Information (ISI) [15,16].

The retrieval method was TS = (soil or field or cropland or mine* or wasteland or industrial) AND (pollution or contamination) AND (heavy metal or lead or chromium or cadmium or zinc or mercury or arsenic or manganese or nickel or iron) AND (removal or remediation* or decontamiation or stabilization or phytoremediation or bioremediation or co-remediation or phytoextraction or physical remediation or carrying soil or soil replacement or thermal treatment or electrokimetic remediation or chemical remediation or leaching or redox or solidification or microbiol remediation or fixation or precipitation).

TS stands for “Topic Subject” search in a Web of Science search. TS retrieval is a Boolean logic-based method of using subject words to search for data, which can quickly and easily obtain a large amount of subject-related data.

The time range is 1999–2020. The data collection time was 30 September 2020. After screening, comparison and weighting, we finally obtained 7332 bibliographic bibliographies. Each bibliography included authors, institutions, abstracts, key words, publication years, issues (volumes) and references. Afterwards, manual methods were used to clean the data, mainly to merge synonyms and delete meaningless words to avoid affecting the analysis results.

### 2.2. Research Methods

Science mapping is a relatively new research method in the field of bibliometric analysis in recent years [17]. It can not only reveal the source of knowledge and its development laws but also graphically express the relationship and evolutionary laws of knowledge structure in related fields. There are many tools for knowledge graph analysis, and each tool has its own advantages and characteristics [18,19]. CiteSpace is a citation network visualization tool developed by Dr. Chen Chaomei of Drexel University in the United States [20,21]. It can draw co-citation maps, keyword maps and time zone views and dynamically identify co-citation clusters, key nodes and research hotspots [22]. Vosviewer is a visualization software developed by Van Eck and Waltman of Leiden University in the Netherlands. It can draw co-occurrence maps of authors, citations, keywords, and other data. This software has unique advantages in clustering technology and map drawing [23,24].

CiteSpace focuses on representing the strength of the relationship between each theme by tree graph and line, while Vosviewer deconstructs the clustering relationship between nodes by distance, density, and other factors. The two can complement each other’s advantages and accurately excavate the essence of the research theme.

For example, a bibliometric analysis of the worldwide scientific publications on CSC was conducted to understand the characteristics and research trends by using CiteSpace [25], comprehensive knowledge review of ER was achieved with the CiteSpace [26], and scientific publications on atlantoaxial spine surgery were analyzed based on Vosviewer [27].

This paper uses CiteSpace and Vosviewer to draw an atlas of scientific knowledge and explore the important citations, themes and frontiers in the research field of soil heavy metal pollution control from 1999 to 2020.

The CiteSpace software was run, and the standardized document title was loaded into the operation. The minimum tree generation algorithm was selected to map the co-cited literature clustering views, journal source, strongest citation bursts list, time zone view and other data.

Using correlation strength, similarity *Sij* between two items *i* and *j* is calculated as

(1) $$S_{ij}=\frac{C_{ij}}{W_{i}W_{j}}$$

where *cij* represents the co-occurrence times of items *i* and *j*, and *Wi* and *Wj* represent the total occurrence times of items *i* and *j* or the total co-occurrence of these items. It can be proven that the similarity between items *i* and *j* calculated using (1) is proportional to the ratio between the observed number of co-occurrences of items *i* and *j* on the one hand and the expected number of co-occurrences on the other hand. Table 1 shows an example of a major term source articel matrix.

An 80 * 80 co-word matrix was constructed, and the standardized similarity matrix was obtained by Ochiai coefficient processing. The matrix was converted into a specified format by Pajek software and imported into the Vosviewer program, and the majorization algorithm of node correlation intensity was selected to generate the co-occurrence keyword clustering view and density view, showing the research organization.

## 3. Results and Discussion

### 3.1. Chronological Distribution

A total of 7587 publications related to heavy metal-contaminated soil remediation research were obtained from 1999 to 2020. The publications were categorized into six document types (article, review, proceedings paper, editorial material, letter and meeting abstract).

**Figure 1:**
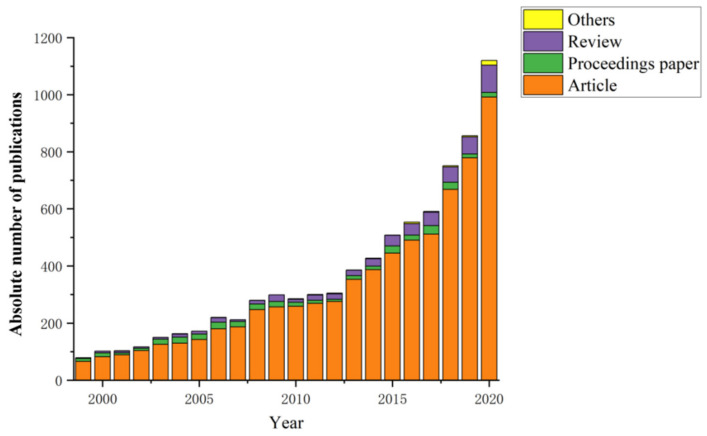
Distribution of publication during 1999–2020.

Figure 1 shows the distribution of publications for all types by year (1999–2020). The document type of article accounted for 89% of the total publications, followed by reviews (6.5%) and proceedings papers (4.6%). Other types, such as letters, editorial material and meeting abstracts, accounted for 0.4% of the total publications.

The chronological distribution of the retrieved publications is shown in Figure 1. According to the analysis of the annual distribution, those years can be divided into two stages: exploratory stage (1999–2012) and rapid development stage (2013–present).

(1) Exploratory stage (1999–2012)

In this stage, the annual number of publications on soil heavy metal restoration was under 300. Over this 14-year period, 2565 papers were published, with a growth rate of 47.4% compared to the figure in 1999. At this stage, there has been increasing interest in finding new and innovative solutions for the efficient removal of contaminants from soils [28,29,30,31].

(2) Rapid development stage (2013-present)

In this stage, the number of papers was 1082 through 2020. The quantity of researchers increased rapidly, with large annual numbers of publications and a rapid growth rate. The progression of knowledge has opened new fields of interest. Metal-organic framework-based materials were used to capture metal ions, and biochar was used for the remediation of soils contaminated with heavy metals and organic pollutants [32,33,34].

Judging from the general trend of Figure 1, the study of soil remediation technologies may continue to flourish in the coming years.

### 3.2. Country and Institution Distribution

**Figure 2:**
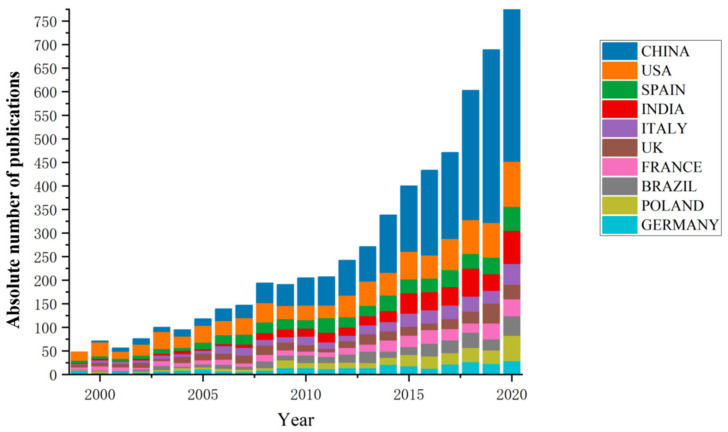
Top 10 countries in soil pollution remediation from 1999 to 2020.

The number of papers issued by each country represents the degree of research activity in the country. From 1999 to 2020, 94 countries or regions published relevant research papers on the remediation of heavy metal-contaminated soil. The specific publication situation of the top 10 countries in terms of publication volume is shown in Figure 2.

China (2173, 28.64%) and the United States (946, 12.47%) are the top two countries in terms of the number of articles published, followed by Spain (476, 6.27%), India (456, 6.01%), Italy (366, 4.82%) and France (339, 5.26%).

**Figure 3:**
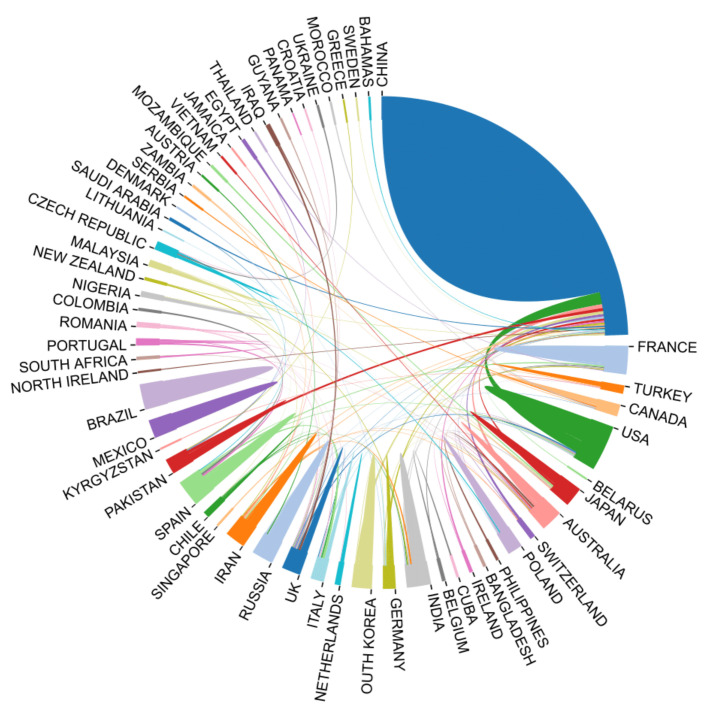
Chord diagram of the 60 most productive countries.

The cooperation relation among the countries in the field of heavy metal-contaminated soil remediation was also analyzed by using social network analysis (see Figure 3). China and the United States played significant and core roles in the cooperation network and had the closest cooperation with greatest number of countries, especially England, Germany and the United States [35].

**Figure 4:**
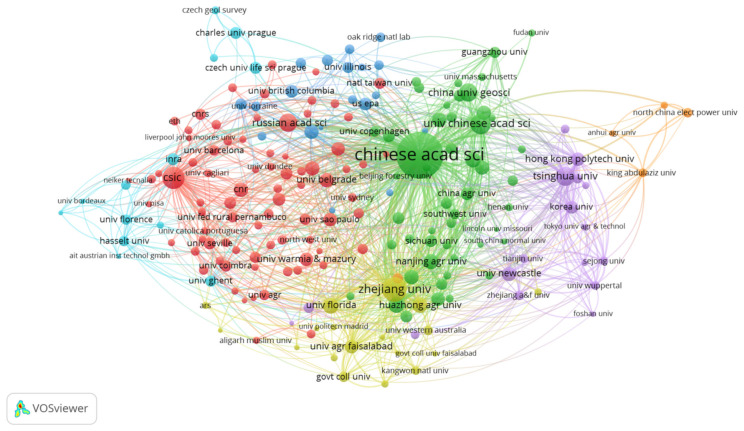
Map of research institutions.

The institution that publishes the paper is subject to the first author. CiteSpace software is used to sort out the institutions of the publications, and the atlas of research institutions is shown in Figure 4. In the figure, a node represents an organization, and the size of the node reflects the number of posts issued by the organization. From 1999 to 2020, the top 15 institutions in the world in terms of the volume of publications on the remediation of heavy metal-contaminated soils are shown in Table 2. Among the top 15 institutions in the world, there were 10 teams from China, which accounted for 40% of the top 15 institutions. The rest were from the United States, Spain, Russia and the UK.

The Chinese Academy of Sciences published 485 articles on heavy metal-contaminated soil remediation technology during 1999–2020. This was much higher than that of the Spanish Council for higher scientific research (CSIC, 111 articles) and Zhejiang University (145 articles). This result showed that China was highly active in this area and that China’s soil pollution problem is relatively serious.

### 3.3. Source Journal Analysis

Analysis of the source journals of a certain area of research can help researchers accurately grasp the core journals in the research field and provide professional guidance for literature queries, data collection, paper writing and contribution [36,37].

Table 3 shows the source analysis of the top 15 journals in terms of heavy metal-contaminated soil remediation technology from 1999 to 2020.

To analyze the documents in the WOS through the application of Vosviewer, the top 15 journals containing research papers related to heavy metal-contaminated soil recovery technology mainly include Environmental Science and Pollution Research, Science of the Total Environment, Chemosphere, Environmental Pollution, and Journal of Hazardous Materials. The top three journals were Environmental Science and Pollution Research (384, 5.06%), Science of the Total Environment (345, 4.55%) and Chemosphere (296, 3.90%). The Journal of Hazardous Materials had the highest average impact factor in the past five years. The research literature published in journals in this field mainly focuses on environmental pollution and phytoremediation and further proves that phytoremediation is the main research direction for current heavy metal-contaminated soil remediation.

### 3.4. Keywords Co-Occurrence Analysis

Keywords are the authors’ highly condensed and generalized core content of the document, which can clearly express the subject of the paper’s research. Based on the statistics and analysis of the subject keywords of the literature, the research hot spots and future research directions of the field can be understood [38,39].

Keywords with high centrality act as important links and have media roles in the keyword network map. The more obvious the centrality, the stronger the control and guiding role in the entire network, indicating that the keyword receives a high degree of attention.

**Figure 5:**
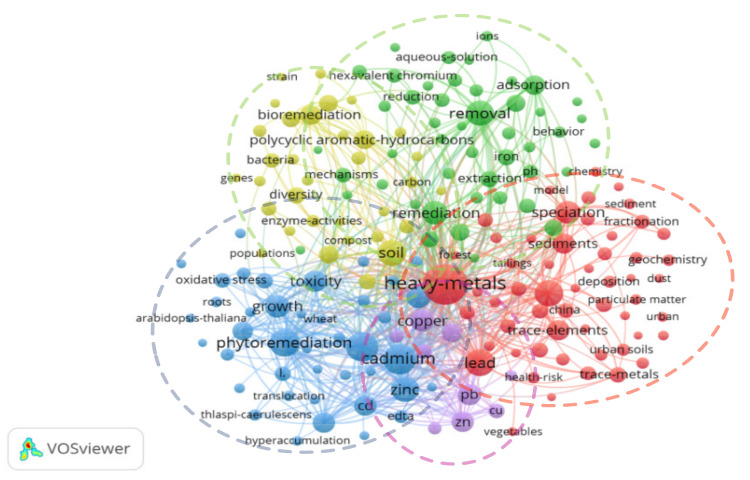
Co-occurrence network of keywords.

Figure 5 shows the overview of the keyword co-occurrence map with 106 nodes. It is clear that five distinct clusters can be found.

The red cluster focused mainly on soil heavy metal pollution and health-risk assessment, and the high-frequency keywords covered heavy metals, agricultural soils, health-risk assessment and lead.

The green cluster was concerned with chemical remediation technology of heavy metal pollution in soil. The high-frequency keywords were as follows: nanoparticles, sorption, decomposition and mechanisms.

The blue cluster presents keywords associated with phytoremediation technology of soil pollution by heavy metals. The high-frequency keywords covered phytoremediation, bioaccumulation and hyperaccumulation.

The yellow cluster indicates the field of plant-microbial joint remediation technology for soil contaminated by heavy metals. The high-frequency keywords included bioremediation, enzyme activities and microorganisms.

The purple cluster represents remediation technology for heavy metal pollution in mines. The high-frequency keywords were as follows: bioavailability and mine tailings.

In the keyword co-occurrence map, the keywords of each cluster are determined by the frequency, and the results are shown in Table 4. These works usually represent the context of heavy metal pollution research.

**Figure 6:**
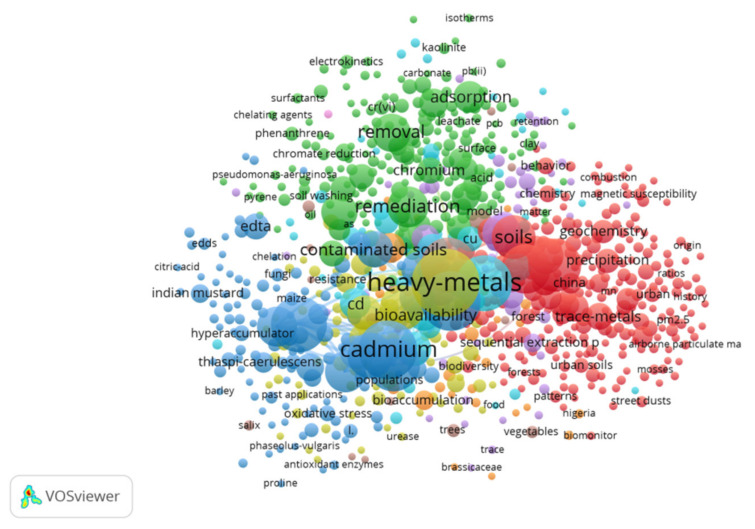
Keywords between 1999 and 2012.

**Figure 7:**
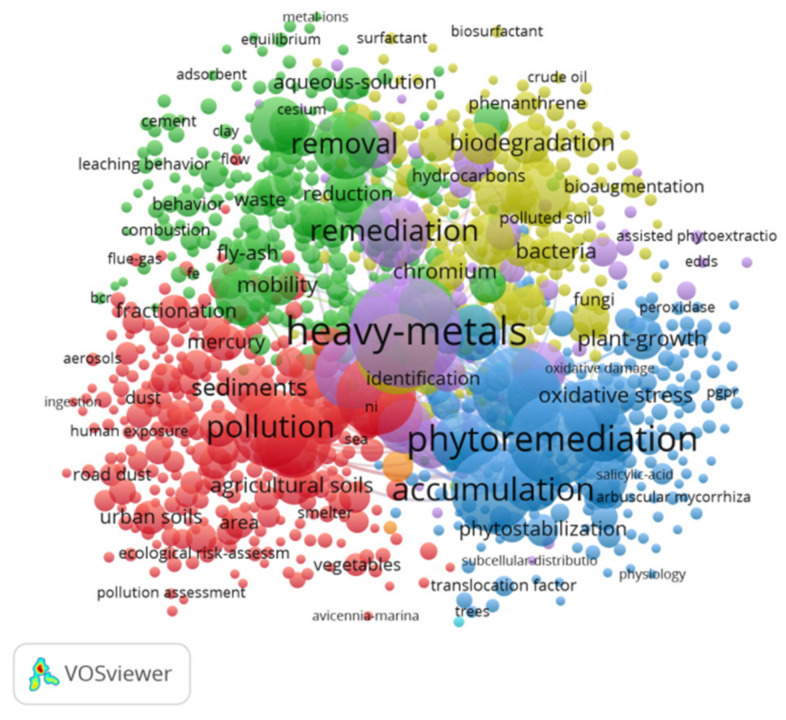
Keywords between 2013 and 2020.

Figure 6 demonstrates the network on heavy metal pollution research from 1999 to 2012. Figure 7 demonstrates the network on heavy metal pollution research from 2013 to 2020. We can see that during 1999–2012, the keywords that appear more frequently are cadmium, bioavailability, removal, and adsorption. During 2013–2020, the keywords that appear more frequently are plant growth, phytoremediation, removal, bioremediation, and biodegradation. After 2013, the research network became more intensive and covered more areas.

### 3.5. Reference Co-Citation Analysis

As shown in Table 5, the critical references of the 19 clusters are displayed visually and chronologically. Figure 7 suggests that plant growth, biochar application and zinc accumulation formed the three largest clusters. Rape cultivar, bacterial community and geochemical fractionation were the three youngest clusters, and feasible soil, in situ soil remediation and nonurban areas were the three oldest clusters. The minimum value of silhouettes was 0.711, and the maximum value of silhouettes was 1.

The largest cluster (#0) had 183 members and a silhouette value of 0.711. The top three most active cited documents in the cluster were ‘Bioremediation of soils contaminated with polycyclic aromatic hydrocarbons, petroleum, pesticides, chlorophenols and heavy metals by composting: Applications, microbes and future research needs’, ‘Using biochar for remediation of soils contaminated with heavy metals and organic pollutants’ and ‘Biological technologies for the remediation of co-contaminated soil’.

Cluster #1 had 163 members and a silhouette value of 0.866. The top three most active cited documents in the cluster were ‘Bioremediation of soils contaminated with polycyclic aromatic hydrocarbons, petroleum, pesticides, chlorophenols and heavy metals by composting: Applications, microbes and future research needs’, ‘Using biochar for remediation of soils contaminated with heavy metals and organic pollutants’ and ‘Biological technologies for the remediation of co-contaminated soil’.

Cluster #2 had 155 members and a silhouette value of 0.833. The top three most active cited documents in the cluster were ‘Phytoremediation of contaminated soils and groundwater: lessons from the field’, ‘Urban environmental geochemistry of trace metals’ and ‘Phytoremediation Technology: Hyperaccumulation of Metals in Plants’.

The most cited references can be used to reflect emerging trends in scientific research. The sudden increase in citations indicates that scholars have paid special attention to the corresponding publications [40,41]. Table 6 lists the top 20 references with the strongest citation bursts.

Yoon J, with an article titled ‘Phytoremediation’, topped the list with an explosive power of 22.73. This article studied the accumulation of Pb, Cu, and Zn in native plants growing on a contaminated Florida site [49]. Phytoremediation can potentially be used to remediate metal-contaminated sites. This study evaluated the potential of 36 plants (17 species) growing on a contaminated site in North Florida. Plants and the associated soil samples were collected and analyzed for total metal concentrations.

Li ZY, with an article titled ‘Pollution and health risk assessment’, ranked second with an explosive power of 21.47. A comprehensive pollution and health risk assessment was conducted in this article [58].

The article by Nagajyoti PC, titled ‘The range of heavy metals’, ranked third with an explosive power of 21.37. This article details the range of heavy metals, their occurrence and toxicity to plants [54].

## 4. Conclusions

This paper conducts a comprehensive bibliometric analysis of the research in the field of soil heavy metal pollution remediation. Based on the number of documents, document sources, keyword clustering and topic analysis, this paper determines the topic framework in this research field. The analysis of the knowledge map shows that the problem of heavy metals in soil has attracted widespread attention from scholars worldwide, the international trend of cooperative research is becoming increasingly obvious, and the trend of multidisciplinary and multitechnology integration is gradually taking shape. China is becoming one of the most important scientific research forces for soil heavy metal research in the world.

In addition, a review of the key themes of soil heavy metal research found that the connotation of soil heavy metal research continues to expand. Based on the study of the source analysis and distribution characteristics of heavy metals in the soil environment to the health risk assessment of heavy metal-contaminated soil and then to the remediation technology of heavy metal pollution, the research context is clear, and the characteristics are obvious. A series of results in the area of soil heavy metal research provide theoretical support and practical guidance for the development of soil remediation work.

Future research on soil heavy metals should be considered in the following contexts: actively carry out international cooperation and technical exchanges, carry out cross-regional and cross-border research, and strive to build a global soil heavy metal pollution remediation network. Scholars from different disciplines, such as environmental science, agronomy, chemistry, geology, materials science, and engineering technology, should be encouraged to jointly develop new soil heavy metal pollution remediation technologies. At the molecular level, we explored the main environmental driving factors of soil heavy metal pollution and explored its interaction mechanisms with enzyme activities and characteristic microorganisms.

The field should actively develop joint remediation technology of heavy metal-contaminated soil, continue to find, screen and cultivate excellent hyperaccumulator plants, and combine the application of molecular biology technology and genetic engineering technology to improve remediation efficiency and reduce costs.

## Figures and Tables

**Table 1:** An example of a major term source article matrix.

No.	Major Terms	PMIDs of Source Article		
		1	2	3
1	soil	0	0	0
2	pollution	0	0	0
3	heavy metal	1	0	0
...	...	...	...	...

**Table 2:** Top 15 research institutions.

Rank	Institution	Country/Region	Amount of Papers/Article	Citations
1	Chinese Acad Sci	CHINA	485	309,762
2	Zhejiang Univ	CHINA	135	101,537
3	CSIC	SPAIN	111	86,641
4	Univ Chinese Acad Sci	CHINA	98	69,877
5	Tsinghua Univ	CHINA	78	63,363
6	China Univ Geosci	CHINA	77	43,091
7	Russian Acad Science	RUSSIA	74	19,270
8	Univ Florida	USA	57	53,040
9	Nothwest A&F Univ	CHINA	57	48,035
10	Huazhong Agr Univ	CHINA	53	42,452
11	Nanjing Univ	CHINA	52	39,840
12	Nanjing Agr Univ	CHINA	50	34,287
13	Beijing Normal univ	CHINA	48	25,135
14	CNR	/	46	19,469
15	Univ nacl autonoma mexico	MEXICO	45	46,465

**Table 3:** Top 15 journal sources of heavy metal pollution soil remediation technologies.

Journal Name	Total Publication	Percentage/%	Impact Factor (2019)	*h*-Index(R)
Environmental Science and pollution research	384	5.06	3.056	98
Science of the Total Environment	345	4.55	6.551	224
Chemosphere	296	3.90	5.778	228
Environmental Pollution	226	2.98	6.792	211
Journal of Hazardous Materials	216	2.85	9.038	260
Water Air and Soil Pollution	178	2.35	1.9	105
Ecotoxicology and Environmental Safety	168	2.21	4.872	121
Environmental Monitoring and Assessment	149	1.96	1.903	102
International Journal of Phytoremediation	148	1.95	2.528	78
Journal of Soils and Sediments	129	1.70	2.763	65
Environmental Earth Sciences	108	1.42	2.18	107
Environmental Science Technology	101	1.33	7.864	373
Journal of Environmental management	100	1.32	5.647	161
Environmental geochemistry and health	90	1.19	3.472	68
Soil sediment contamination	84	1.11	1.25	45

**Table 4:** Top 40 keywords of the publications.

No.	Keyword	Absolute Frequency	No.	Keyword	Absolute Frequency
1	heavy-metals	1925	21	bioavailability	417
2	phytoremediation	1253	22	adsorption	416
3	cadmium	1195	23	cd	390
4	soil	1031	24	sediments	382
5	pollution	952	25	zn	364
6	accumulation	939	26	trace-elements	360
7	lead	932	27	immobilization	287
8	contamination	875	28	Polycyclic aromatic-hydrocarbons	284
9	remediation	718	29	biodegradation	253
10	zinc	693	30	sorption	252
11	removal	633	31	arsenic	238
12	bioremediation	562	32	degradation	237
13	plants	548	33	mobility	228
14	copper	543	34	extraction	227
15	phytoextraction	499	35	organic-matter	225
16	speciation	499	36	iron	224
17	contaminated soils	464	37	diversity	208
18	growth	460	38	chromium	206
19	toxicity	451	39	bacteria	203
20	pb	438	40	mercury	201

**Table 5:** Major cluster of co-cited references.

Cluster ID	Size	Silhouette	Label	Mean (Cite Year)
0	183	0.711	Plant growth	2011
1	163	0.866	Biochar application	2013
2	155	0.833	Zinc accumulation	2004
3	145	0.815	Sw spain	2003
4	118	0.858	Health risk	2013
5	107	0.944	Zea may	1999
6	90	0.869	Helianthus annuus	2007
7	76	0.964	Feasible soil	1996
8	63	0.887	Rape cultivar	2016
9	62	0.964	Magnetic susceptibility measure	2005
10	43	0.946	Bacterial community	2014
11	35	0.997	Situ soil remediation	1996
13	21	0.974	Metal ion release	2001
14	20	0.983	Interactive effect	2008
15	18	0.998	Non-urban area	1998
17	14	0.989	Geochemical fractionation	2017
21	8	0.999	Cleveland area commom	2000
24	5	0.999	Pollution source	2006
28	3	1	Waste incineration residue	2010

**Table 6:** Top 20 references with the strongest citation bursts.

References	Strength	Begin	End	1999–2020
Giller K.E. [42]	15.98	1999	2020	▃▃▃▃▃▃▃▃▃▃▃▃▃▃▃▃▃▃▃▃▃▃
Salt D.E. [43]	15.68	2000	2020	▂ ▃▃▃▃▃▃▃▃▃▃▃▃▃▃▃▃▃▃▃▃▃
Adriano D.C. [44]	20.96	2004	2020	▂▂ ▂▂▂ ▃▃▃▃▃▃▃▃▃▃▃▃▃▃▃▃▃
Kabata P.A. [45]	20.29	2005	2020	▂▂ ▂▂▂▂ ▃▃▃▃▃▃▃▃▃▃▃▃▃▃▃▃
Pulford I.D. [46]	18.73	2005	2020	▂▂▂▂ ▂▂ ▃▃▃▃▃▃▃▃▃▃▃▃▃▃▃▃
Ma L.Q. [47]	17.72	2005	2020	▂▂ ▂▂▂▂ ▃▃▃▃▃▃▃▃▃▃▃▃▃▃▃▃
Adriano D.C. [48]	15.79	2006	2020	▂▂▂▂▂ ▂▂ ▃▃▃▃▃▃▃▃▃▃▃▃▃▃▃
Yoon J. [49]	22.73	2010	2020	▂▂▂▂▂▂▂ ▂▂▂▂ ▃▃▃▃▃▃▃▃▃▃▃
Vangronsveld J. [50]	20.58	2011	2020	▂▂▂▂▂▂▂▂▂▂ ▂▂ ▃▃▃▃▃▃▃▃▃▃
Kumpiene J. [51]	19.26	2011	2020	▂▂▂▂▂▂▂▂▂ ▂▂▂ ▃▃▃▃▃▃▃▃▃▃
Glick B.R. [52]	16.22	2012	2020	▂▂▂▂▂▂▂▂▂▂▂ ▂▂ ▃▃▃▃▃▃▃▃▃
Wei B.G. [53]	17.01	2013	2020	▂▂▂▂▂▂▂▂▂▂▂ ▂▂▂ ▃▃▃▃▃▃▃▃
Nagajyoti P.C. [54]	21.37	2015	2020	▂▂▂▂▂▂▂▂▂▂▂ ▂▂▂▂▂ ▃▃▃▃▃▃
Wuana R.A. [55]	14.57	2015	2020	▂▂▂▂▂▂▂▂▂▂▂▂ ▂▂▂▂ ▃▃▃▃▃▃
Ali H. [56]	17.66	2016	2020	▂▂▂▂▂▂▂▂▂▂▂▂▂▂ ▂▂▂ ▃▃▃▃▃
Bolan N. [57]	14.26	2017	2020	▂▂▂▂▂▂▂▂▂▂▂▂▂▂▂ ▂▂▂ ▃▃▃▃
Li Z.Y. [58]	21.47	2018	2020	▂▂▂▂▂▂▂▂▂▂▂▂▂▂▂ ▂▂▂▂ ▃▃▃
Mahar A. [59]	21.11	2018	2020	▂▂▂▂▂▂▂▂▂▂▂▂▂▂▂▂▂ ▂▂ ▃▃▃
Khalid S. [60]	17.91	2018	2020	▂▂▂▂▂▂▂▂▂▂▂▂▂▂▂▂▂▂ ▂ ▃▃▃
Sarwar N. [61]	16.16	2018	2020	▂▂▂▂▂▂▂▂▂▂▂▂▂▂▂▂▂▂ ▂ ▃▃▃

## Data Availability

No applicable.

## References

[1] Armah F.A., Obiri S., Yawson D.O., Onumah E.E., Yengoh G.T., Afrifa E.K.A., Odoi J.O. (2010). Anthropogenic Sources and Environmentally Relevant Concentrations of Heavy Metals in Surface Water of a Mining District in Ghana: A Multivariate Statistical Approach. J. Environ. Sci. Health Part A.

[2] Han R., Zhou B., Huang Y., Lu X., Li S., Li N. (2020). Bibliometric Overview of Research Trends on Heavy Metal Health Risks and Impacts in 1989–2018. J. Clean. Prod..

[3] Li N., Han R., Lu X. (2018). Bibliometric Analysis of Research Trends on Solid Waste Reuse and Recycling during 1992–2016. Resour. Conserv. Recycl..

[4] Bori J., Vallès B., Navarro A., Riva M.C. (2017). Ecotoxicological Risks of the Abandoned F–Ba–Pb–Zn Mining Area of Osor (Spain). Environ. Geochem. Health.

[5] Liu S., Wang X., Guo G., Yan Z. (2021). Status and Environmental Management of Soil Mercury Pollution in China: A Review. J. Environ. Manag..

[6] Tóth G., Hermann T., Szatmári G., Pásztor L. (2016). Maps of Heavy Metals in the Soils of the European Union and Proposed Priority Areas for Detailed Assessment. Sci. Total Environ..

[7] Obrist D., Kirk J., Zhang L., Sunderland E., Jiskra M., Selin N. (2018). A Review of Global Environmental Mercury Processes in Response to Human and Natural Perturbations: Changes of Emissions, Climate, and Land Use. Ambio.

[8] Brunetti G., Farrag K., Soler-Rovira P., Ferrara M., Nigro F., Senesi N. (2012). The Effect of Compost and Bacillus Licheniformis on the Phytoextraction of Cr, Cu, Pb and Zn by Three Brassicaceae Species from Contaminated Soils in the Apulia Region, Southern Italy. Geoderma.

[9] Hema T.G., Getha K., Tan G.Y.A., Sahira H.L., Syamil A.M., Fairuz M.Y.N. (2014). Actionbacteria isolates from tin tailings and forest soil for bioremediation of heavy metals. J. Trop. For. Sci..

[10] Ubando A.T., Africa A.D.M., Maniquiz-Redillas M.C., Culaba A.B., Chen W.-H., Chang J.-S. (2021). Microalgal Biosorption of Heavy Metals: A Comprehensive Bibliometric Review. J. Hazard. Mater..

[11] van Nunen K., Li J., Reniers G., Ponnet K. (2018). Bibliometric Analysis of Safety Culture Research. Saf. Sci..

[12] Zhang W., Zhang Q., Yu B., Zhao L. (2015). Knowledge Map of Creativity Research Based on Keywords Network and Co-Word Analysis, 1992–2011. Qual. Quant..

[13] Luo R., Li J., Zhao Y., Fan X., Zhao P., Chai L. (2017). A Critical Review on the Research Topic System of Soil Heavy Metal Pollution Bioremediation Based on Dynamic Co-Words Network Measures. Geoderma.

[14] Zhou W., Kou A., Chen J., Ding B. (2018). A Retrospective Analysis with Bibliometric of Energy Security in 2000–2017. Energy Rep..

[15] Cui T., Zhang J. (2018). Bibliometric and Review of the Research on Circular Economy through the Evolution of Chinese Public Policy. Scientometrics.

[16] Bartol T., Mackiewicz-Talarczyk M. (2015). Bibliometric Analysis of Publishing Trends in Fiber Crops in Google Scholar, Scopus, and Web of Science. J. Nat. Fibers.

[17] Chiu W.-T., Ho Y.-S. (2007). Bibliometric Analysis of Tsunami Research. Scientometrics.

[18] Zhang Y., Huang K., Yu Y., Yang B. (2017). Mapping of Water Footprint Research: A Bibliometric Analysis during 2006–2015. J. Clean. Prod..

[19] Hood W.W., Wilson C.S. (2001). The Literature of Bibliometrics, Scientometrics, and Informetrics. Scientometrics.

[20] Chen C. (2006). CiteSpace II: Detecting and Visualizing Emerging Trends and Transient Patterns in Scientific Literature. J. Am. Soc. Inf. Sci. Technol..

[21] Chen C., Ibekwe-Sanjuan F., Hou J. (2014). The Structure and Dynamics of Co-Citation Clusters: A Multiple-Perspective Co-Citation Analysis. J. Am. Soc. Inf. Technol..

[22] Chen C.C., Wu C.F., Yu H.L., Chan C.C., Cheng T.J. (2012). Spatiotemporal Modeling with Temporal-Invariant Variogram Subgroups to Estimate Fine Particulate Matter PM2.5 Concentrations. Atmos. Environ..

[23] Pan X., Yan E., Cui M., Hua W. (2018). Examining the Usage, Citation, and Diffusion Patterns of Bibliometric Mapping Software: A Comparative Study of Three Tools. J. Informetr..

[24] van Eck N.J., Waltman L. (2010). Software Survey: VOSviewer, a Computer Program for Bibliometric Mapping. Scientometrics.

[25] Yang F., Qiu D. (2019). Exploring coal spontaneous combustion by bibliometric analysis. Process. Saf. Environ. Prot..

[26] Yang R., Wong C., Miao M. (2021). Analysis of the trend in the knowledge of environmental responsibility research. J. Clean. Prod..

[27] Xie L., Chen Z., Wang H. (2020). Bibliometric and Visualized Analysis of Scientific Publications on Atlantoaxial Spine Surgery Based on Web of Science and VOSviewer. World Neurosurg..

[28] Wang J., Chen C. (2006). Biosorption of Heavy Metals by Saccharomyces Cerevisiae: A Review. Biotechnol. Adv..

[29] Liu X., Gao Y., Khan S., Duan G., Chen A., Ling L., Zhao L., Liu Z., Wu X. (2008). Accumulation of Pb, Cu, and Zn in Native Plants Growing on Contaminated Sites and Their Potential Accumulation Capacity in Heqing, Yunnan. J. Environ. Sci..

[30] Gadd G.M. (2010). Biosorption: Critical Review of Scientific Rationale, Environmental Importance and Significance for Pollution Treatment. J. Chem. Technol. Biotechnol..

[31] Beesley L., Moreno-Jimenez E., Gomez-Eyles J.L., Harris E., Robinson B., Sizmur T. (2011). A Review of Biochars’ Potential Role in the Remediation, Revegetation and Restoration of Contaminated Soils. Environ. Pollut..

[32] Ali H., Khan E., Sajad M.A. (2013). Phytoremediation of Heavy Metals—Concepts and Applications. Chemosphere.

[33] Ahmad M., Rajapaksha A.U., Lim J.E., Zhang M., Bolan N., Mohan D., Vithanage M., Lee S.S., Ok Y.S. (2014). Biochar as a Sorbent for Contaminant Management in Soil and Water: A Review. Chemosphere.

[34] Li J., Wang X., Zhao G., Chen C., Chai Z., Alsaedi A., Hayat T., Wang X. (2018). Metal–Organic Framework-Based Materials: Superior Adsorbents for the Capture of Toxic and Radioactive Metal Ions. Chem. Soc. Rev..

[35] Hosseini M.R., Martek I., Zavadskas E.K., Aibinu A.A., Arashpour M., Chileshe N. (2018). Critical Evaluation of Off-Site Construction Research: A Scientometric Analysis. Autom. Constr..

[36] Su H.N., Lee P.C. (2010). Mapping Knowledge Structure by Keyword Co-Occurrence: A First Look at Journal Papers in Technology Foresight. Entometrics.

[37] Hu L., Zhang L., Wu H. (2018). Experimental study of the effects of soil pH and ionic species on the electro-osmotic consolidation of kaolin. J. Hazard. Mater..

[38] Oraee M., Hosseini M.R., Papadonikolaki E., Palliyaguru R., Arashpour M. (2017). Collaboration in BIM-Based Construction Networks: A Bibliometric-Qualitative Literature Review. Int. J. Proj. Manag..

[39] Zhang Q., Feng Q., Zhu X., Zhang M., Wang Y., Yang L. (2021). Box Experiment Study of Thermally Enhanced SVE for Benzene. Int. J. Environ. Res. Public Health.

[40] Xia W., Du Y., Li F., Li C., Yan X., Arul A., Wang F., Song D. (2019). In-situ solidification/stabilization of heavy metals contaminated site soil using a dry jet mixing method and new hydroxyapatite based binder. J. Hazard. Mater..

[41] Li C., Ji X., Luo X. (2019). Phytoremediation of Heavy Metal Pollution: A Bibliometric and Scientometric Analysis from 1989 to 2018. Int. J. Environ. Res. Public Health.

[42] Giller K.E., Witter E., Mcgrath S.P. (1998). Toxicity of Heavy Metals to Microorganisms and Microbial Processes in Agricultural Soils: A Review. Soil Biol. Biochem..

[43] Salt D.E., Smith R.D., Raskin I. (1998). Phytoremediation. Annu. Rev. Plant Physiol. Plant Mol. Biol..

[44] Adriano D.C., Adriano D.C. (2001). Arsenic. Trace Elements in Terrestrial Environments: Biogeochemistry, Bioavailability, and Risks of Metals.

[45] Kabata-Pendias A. (2004). Soil–Plant Transfer of Trace Elements—An Environmental Issue. Geoderma.

[46] Pulford I.D., Watson C. (2003). Phytoremediation of Heavy Metal-Contaminated Land by Trees—A Review. Environ. Int..

[47] Ma L.Q., Komar K.M., Tu C., Zhang W., Cai Y., Kennelley E.D. (2001). A Fern That Hyperaccumulates Arsenic. Nature.

[48] Adriano D.C., Wenzel W.W., Vangronsveld J., Bolan N.S. (2004). Role of Assisted Natural Remediation in Environmental Cleanup. Geoderma.

[49] Yoon J., Cao X., Zhou Q., Ma L.Q. (2006). Accumulation of Pb, Cu, and Zn in Native Plants Growing on a Contaminated Florida Site. Sci. Total Environ..

[50] Vangronsveld J., Herzig R., Weyens N., Boulet J., Adriaensen K., Ruttens A., Thewys T., Vassilev A., Meers E., Nehnevajova E. (2009). Phytoremediation of Contaminated Soils and Groundwater: Lessons from the Field. Environ. Sci. Pollut. Res..

[51] Kumpiene J., Lagerkvist A., Maurice C. (2008). Stabilization of As, Cr, Cu, Pb and Zn in Soil Using Amendments—A Review. Waste Manag..

[52] Glick B.R. (2010). Using Soil Bacteria to Facilitate Phytoremediation. Biotechnol. Adv..

[53] Wei B., Yang L. (2010). A Review of Heavy Metal Contaminations in Urban Soils, Urban Road Dusts and Agricultural Soils from China. Microchem. J..

[54] Nagajyoti P.C., Lee K.D., Sreekanth T.V.M. (2010). Heavy Metals, Occurrence and Toxicity for Plants: A Review. Environ. Chem. Lett..

[55] Wuana R.A., Okieimen F.E. (2011). Heavy Metals in Contaminated Soils: A Review of Sources, Chemistry, Risks and Best Available Strategies for Remediation. ISRN Ecol..

[56] Ali H., Khan E. (2021). Bioaccumulation of selected toxic heavy metals in mastacembelus armatus from three rivers of Malakand division, Pakistan. Pak. J. Zool..

[57] Bolan N., Kunhikrishnan A., Thangarajan R., Kumpiene J., Park J., Makino T., Kirkham M.B., Scheckel K. (2014). Remediation of Heavy Metal(Loid)s Contaminated Soils—To Mobilize or to Immobilize?. J. Hazard. Mater..

[58] Li Z., Ma Z., van der Kuijp T.J., Yuan Z., Huang L. (2014). A Review of Soil Heavy Metal Pollution from Mines in China: Pollution and Health Risk Assessment. Sci. Total Environ..

[59] Mahar A., Wang P., Ali A., Awasthi M.K., Lahori A.H., Wang Q., Li R., Zhang Z. (2016). Challenges and Opportunities in the Phytoremediation of Heavy Metals Contaminated Soils: A Review. Ecotoxicol. Environ. Saf..

[60] Khalid S., Shahid M., Niazi N.K., Murtaza B., Bibi I., Dumat C. (2017). A Comparison of Technologies for Remediation of Heavy Metal Contaminated Soils. J. Geochem. Explor..

[61] Sarwar N., Imran M., Shaheen M.R., Ishaque W., Kamran M.A., Matloob A., Rehim A., Hussain S. (2017). Phytoremediation Strategies for Soils Contaminated with Heavy Metals: Modifications and Future Perspectives. Chemosphere.

